# Stent Retriever Thrombectomy of Small Caliber Intracranial Vessels Using pREset LITE: Safety and Efficacy

**DOI:** 10.1007/s00062-016-0497-0

**Published:** 2016-01-21

**Authors:** W. Kurre, M. Aguilar-Pérez, R. Martinez-Moreno, E. Schmid, H. Bäzner, H. Henkes

**Affiliations:** 1 Neurologische Klinik, Klinikum Stuttgart, Kriegsbergstrasse 60, 70174 Stuttgart, Germany; 2 Medizinische Fakultät der Universität Duisburg-Essen, Hufelandstrasse 55, 45122 Essen, Germany

**Keywords:** Stroke, Treatment, Endovascular procedures, Thrombectomy

## Abstract

**Purpose:**

Few devices are approved for thrombectomy of distal vessel branches, and clinical experience is limited. Here we report our experience with pREset LITE for thrombectomy of small intracranial vessels.

**Methods:**

From an institutional database we selected consecutive patients treated with pREset LITE for an occlusion of small (≤ 2 mm), intracranial target vessels. Recanalization success was measured by applying the modified Thrombolysis In Cerebral Infarction (mTICI) score. To assess safety, we recorded device-related procedural events and potentially device-related hemorrhages on follow-up imaging. Infarcts in the dependent territory served as a measure for efficacy.

**Results:**

Of 536 patients treated between August 2013 and March 2015, 76 met the inclusion criteria. pREset LITE was used in 90 branches with an average diameter of 1.6 mm (1.3–2.0 mm). An mTICI score ≥ 2b was achieved in 70.0 %. Procedural events consisted of 5.6 % significant vasospasm, 2.2 % suspected dissections, 2.2 % downstream emboli, and 1.1 % self-limiting extravasations. On posttreatment imaging 2.2 % parenchymal hemorrhages type I (PHI) and 13.3 % focal subarachnoid hemorrhage (SAH) were potentially device related, but all of these events remained asymptomatic. After successful recanalization, 33.3 % developed no ischemia in the dependent territory while 41.7 % developed a partial infarct, and 25 % developed a complete infarct. Successful recanalization significantly increased the chance to develop no or only partial infarct compared with a complete infarction (*p* = 0.003, *p* = 0.013).

**Conclusions:**

Thrombectomy of small vessels with pREset LITE is feasible with good recanalization and reasonable safety margins. Successful recanalization significantly reduces the risk of infarction in the dependent territory. The impact on the overall clinical outcome remains to be determined.

## Introduction

Endovascular recanalization of large vessel occlusion with stent retrievers was recently established as an evidence-based therapy for acute stroke. Five randomized trials clearly demonstrated that endovascular treatment in combination with standard medical measures is superior to medical treatment alone [1–5]. Target vessels within these trials were largely confined to the most proximal segments of brain-supplying arteries, which are most likely to cause severe stroke syndromes in case of an acute occlusion and are least likely to be injured by mechanical manipulation. This is in line with the approval of most stent retrievers starting from a vessel diameter of 2 mm and up to 9 mm for the largest available device. Embolic occlusion of vessels smaller than 2 mm is frequently encountered during mechanical thrombectomy and may reflect either extension of a thrombus of a larger vessel into a smaller branch, primary occlusion of a small branch, or spread of thrombus material due to fragmentation during the procedure. If branches supplying eloquent brain tissue are affected, recanalization is desirable. A few new generation stent retrievers with either reduced radial force or small crossing profile or both are now approved for the treatment of vessels with a diameter of > 1.5 mm. Up to now the knowledge about safety and efficacy of thrombectomy in small vessels is limited. Here we report our experience with pREset LITE (phenox GmbH, Bochum, Germany) for the treatment of vessels ≤ 2 mm.

## Methods

### Patient Selection

From a prospectively maintained institutional database we selected consecutive patients treated with pREset LITE in target vessels of ≤ 2 mm diameter between August 2013 and March 2015.

### General Treatment Protocol

Patients referred for mechanical recanalization had a National Institute of Health Stroke Scale (NIHSS) ≥ 4 caused by a major intracranial vessel occlusion of the terminal carotid artery, the M1 or M2 segment of the middle cerebral artery, the basilar artery, or the P1 segment of the posterior cerebral artery. For definition of M2 we used the clinical approach considering the postbifurcational segment as the proximal part of the M2 branch. In multiple vessel occlusions we also treated anterior cerebral artery targets. Fluctuating clinical symptoms were the reason for treatment of patients with minor stroke severity at presentation (NIHSS < 4). Treatment was performed within 8 h from symptom onset. Exceptions were made for patients beyond this time window in case of a mismatch between severity of symptoms and infarct size in imaging studies or fluctuating/progressive symptoms suggesting a salvageable penumbra supplied by collaterals. Patients with unknown time window were also selected based on this concept of clinical mismatch. Computer tomography (CT) or magnetic resonance imaging (MRI) was used as baseline imaging according to the local standards of the referring hospital. Large-vessel occlusion was confirmed by CT angiography (CTA) or magnetic resonance angiography (MRA).

Intravenous Alteplast was given prior to the endovascular procedure in a subset of patients according to the decision of the referring neurologist. Patients with a suspected stenosis or dissection received a loading dose of 500 mg acetylsalicylic acid and 180 mg ticagrelor or 600 mg clopidogrel but no Alteplast to reduce the risk of hemorrhage. If a patient did not receive preloading but stenting had to be performed, 500 mg acetylsalicylic acid was applied intravenously followed by 180 mg ticagrelor or 600 mg clopidogrel via a nasogastric tube. A body weight adapted bolus of eptifibatide was applied intravenously to bridge the period of resorption and activation of the P2Y12 receptor antagonist.

Procedures were routinely performed under general anesthesia by four experienced interventional neuroradiologists. In the anterior circulation an 8 French guide catheter (Guider Softip^TM^ XF, Boston Scientific, Plymouth, MN, USA) was used in combination with an intermediate catheter (5max ACE^TM^, Penumbra Inc., Alameda, CA, USA; Navien A^+^ 0.58^TM^, Medtronic, Dublin, Ireland). A balloon guide catheter (Cello^TM^ 8F, Medtronic, Dublin, Ireland) was rarely chosen. Vascular access in the posterior circulation in large-lumen vertebral arteries was gained with an 8 F guide catheter (Guider Softip^TM^ XF) in combination with an intermediate catheter (5max ACE^TM^, Navien A^+^ 0.58^TM^). In small lumen vertebral arteries a 6 F guide catheter (Envoy XB^TM^, Codman and Shurtleff, Raynham, MA, USA) without an intermediate catheter was chosen. In case of difficult transfemoral access a large lumen intermediate catheter (Navien A^+^ 0.72^TM^, Medtronic, Dublin, Ireland) was introduced transbrachially without a guide catheter. Access vessel stenoses or occlusions were treated by stent angioplasty preferentially prior to the intracranial recanalization procedure.

The occluded target vessel was catheterized with a 0.021- or 0.025-inch inner lumen microcatheter (Trevo^®^ pro 18, Stryker, Mountain View, CA, USA; PX Velocity, Penumbra Inc., Alameda, CA, USA) guided by a 0.014-inch guide wire (Synchro^2 ®^, Stryker Neurovascular, Freemont, CA, USA). For catheterization of small caliber branches either the same microcatheter or a 0.017-inch microcatheter (Echelon^TM^ 10 or 14, Medtronic, Dublin, Ireland) was chosen. The stent retriever (pREset^TM^/pREset LITE^TM^, phenox GmbH, Bochum, Germany; Solitaire^TM^, Medtronic, Dublin, Ireland; Embotrap^TM^, Neuravi, Galway, Ireland; 3D Separator, Penumbra Inc., Alameda, CA, USA) was deployed beyond the assumed occlusion site. After 5-min incubation and intra-arterial injection of 0.5–1 mg glycerol trinitrate the intermediate catheter was navigated as close to the thrombus as possible. During recanalization of M2 segments the intermediate catheter was positioned in the distal M1 segment during recanalization of anterior cerebral artery branches in the distal ICA and during recanalization of posterior cerebral arteries in the distal basilar artery. The thrombectomy device was withdrawn under continuous aspiration of the intermediate catheter. Gentle pulling of the intermediate catheter prevented uncontrolled forward movement during thrombectomy. Arterial hypotension was pharmacologically compensated by intravenous injection of cafedrine hydrochloride/theodrenaline hydrochloride. In case of persistent occlusion or incomplete recanalization, thrombectomy was repeated with the same or another device. If recanalization was not achieved after several thrombectomy maneuvers, the procedure was either aborted or continued using angioplasty and/or stent deployment or intra-arterial thrombolysis as a rescue maneuver.

The decision to treat a small vessel branch during thrombectomy was made by the operator considering the severity and duration of clinical symptoms, collateral status, accessibility of the branch, and eloquence of the dependent brain tissue. pREset LITE was chosen as a device in anatomical situations in which according to the judgment of the operator the use of a regular 4-mm stent retriever appeared to harbor an increased risk considering the vessel diameter, its angulation, and the retraction pathway.

After thrombectomy the patient was kept sedated and ventilated until the next day to allow for precise blood pressure management tolerating a maximum peak systolic value of 130 mm Hg.

Follow-up imaging was done at least 24–48 h posttreatment using either CT or MRI. Further imaging was performed depending on clinical demands.

### pREset LITE Device Design and Assessment

pREset LITE was developed for recanalization of small caliber intracranial vessels (> 1.5 mm) and is officially approved for this purpose in Europe (Conformité Européenne). The device is available with a diameter of 3 and 4 mm. Working length is 20 mm for both versions. Both devices are designed to fit inside a 0.0165-inch microcatheter. The radial force of pREset LITE is significantly lower compared with the regular pREset while cell configuration and overall device design are similar. According to the manufacturer the radial force of pREset LITE 4–20 in a 2-mm vessel is approximately 30 % and that of pREset LITE 3–20 65 % lower compared with a regular pREset 4–20.

### Data Assessment

For all patients we assessed baseline clinical data including age, etiology of stroke, common vascular risk factors, time from symptom onset to treatment, NIHSS at presentation, primary target vessels for treatment, and the rate of successful recanalization (modified thrombolysis in cerebral infarction score (mTICI) 2b or 3). We also reported whether cervical or intracranial artery stenting was performed during the procedure. Ninety-day modified Ranking Scale (mRS) scores were collected for assessment of clinical outcome.

To evaluate the performance of pREset LITE we identified the small caliber target branch the device was used in. Feasibility was defined as successful deployment of pREset LITE at the intended location, and recanalization success was measured applying the mTICI score focused on the target branch. Successful recanalization was defined as mTICI ≥ 2b. Safety aspects were addressed by reporting device-associated adverse events and hemorrhages on follow-up imaging. Hemorrhages were divided into parenchymal hemorrhages type I and II (PHI, PHII) according to the “European Cooperative Acute Stroke Study” and subarachnoid hemorrhage (SAH) [6]. A hemorrhage was classified as possibly device related if it was anatomically related to the target branch and no other cause was identifiable. A device-related adverse event or hemorrhage was regarded as symptomatic if the NIHSS increased by ≥ 4 points, and the adverse event or hemorrhage was the most likely cause for the clinical deterioration. Follow-up imaging was also evaluated regarding new infarcts in the territory of the target branch and findings categorized into no, partial, and complete infarction. If more than one follow-up examination existed, all were screened for hemorrhage and infarction.

### Statistics

Fisher’s exact test was used to assess the dependency between recanalization of the target branch and infarction on follow-up imaging, in general, as well as the influence of the recanalization status on the extent of infarction and the influence of occlusion type on the extent of infarction. A *p*-value < 0.05 was defined as statistically significant. Odds ratio (OR) and 95 % confidence intervals (95 % CI) were calculated for the influence of the recanalization status on the extent of infarction. All analyses were performed with STATA/IC 11.2 for Windows software (StataCorp, College Station, Texas).

## Results

**Fig. 1:**
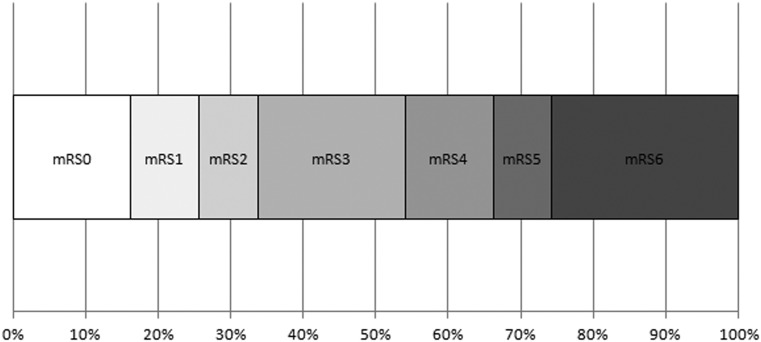
Distribution of mRS scores at 90 days (data missing for two patients)

In the predefined period 536 patients were treated by endovascular approach for acute ischemic stroke. Of these 76 fulfilled the inclusion criteria. Baseline clinical data are summarized in Table 1. Primary targets for treatment were the terminal carotid artery (*n* = 17), the M1 segment of the middle cerebral artery (MCA) (*n* = 31), the M2 segment of the MCA (*n* = 20), the pericallosal (*n* = 2), basilar (*n* = 4), or posterior cerebral artery (*n* = 5). Three patients had two targets each. Distal access catheters were used in 74 patients (97.4 %), in combination with a regular guide catheter in 69 (90.8 %) and in combination with a balloon guide in five (6.5 %). A regular guide catheter without distal access catheter was used in two patients (2.6 %). Successful recanalization was achieved in 69 targets (87.3 %). Cervical artery stenting was performed in 16 patients (21.1 %), and 9 received intracranial stents (11.8 %). The mRS at 90 days is summarized in Fig. 1.

**Table 1:** Baseline clinical data of patients treated with pREset LITE

Number of patients (*n* )	76
Average age (years (range))	71 (36–93)
Female (*n* (%))	34 (44.7 %)
Average NIHSS at presentation (range)^a^	14 (0–27)
Average time from onset to treatment (min (range))	255 (112–486)
Unknown time of clinical onset (*n* = (%))	17 (22.4 %)
Progressive or fluctuating symptoms (*n* = (%))	4 (5.3 %)
Treatment more than 8 h after symptom onset	5 (6.6 %)
**Stroke etiology (** ***n*** **(%))**	
Cardiac embolism	46 (60.5 %)
Extra- or intracranial large artery atherosclerosis	14 (18.4 %)
Embolic stroke of unknown etiology	7 (9.2 %)
Rare causes^b^	9 (11.8 %)
**Cardiovascular risk factors (** ***n*** **(%))**	
Atrial fibrillation	50 (65.8 %)
Diabetes mellitus	18 (23.7 %)
Hypercholesterolemia	22 (28.9 %)
Hypertension	58 (76.3 %)
Current smoker	13 (17.1 %)
Coronary artery disease	19 (25.0 %)
Peripheral artery disease	8 (10.5 %)

^a^Available for *n* = 68 (five patients were referred intubated and in three patients the NIHSS was not assessed)

^b^Includes dissection, endocarditis, paraneoplastic disease, stent thrombosis, and iatrogenic stroke during surgical or endovascular procedures

**Fig. 2:**
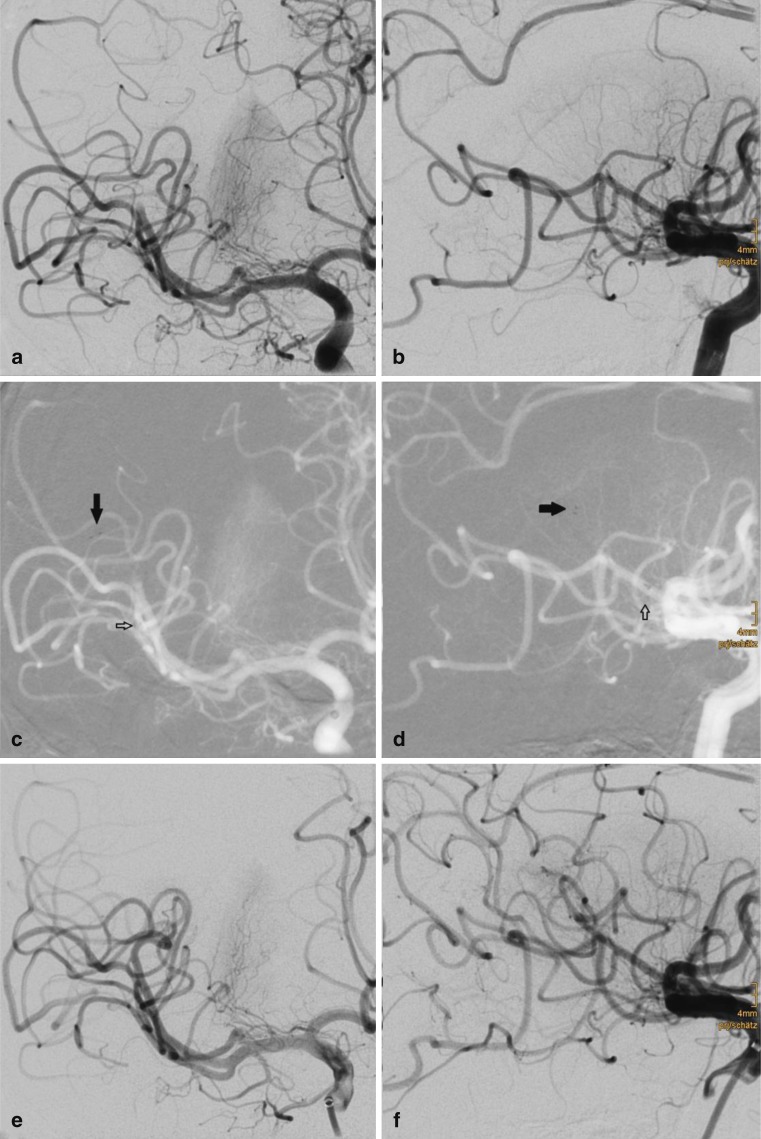
The patient was referred for the treatment of a right M1 occlusion after failure of intravenous thrombolysis. After the first pass partial recanalization was achieved, but an embolic occlusion of the angular artery occurred (**a, b**). A pREset LITE 4–20 was deployed starting in the M3 segment with the proximal landing zone in M2 (**c, d**, *arrow* = distal markers, open *arrow* = proximal marker). A TICI 3 result was achieved after thrombectomy (**e, f**)

Recanalization of small caliber branches using pREset LITE was attempted in 90 targets. Of the latter 63 were primary occlusions, 14 were emboli into the already affected territory, 12 were emboli into a new territory, and 1 was undetermined. A typical case is presented in Fig. 2. Location of the target branches, average target vessel diameters, recanalization results after thrombectomy with pREset LITE, and the number of passes are summarized in Table 2. Successful recanalization was achieved in 63 targets (70.0 %) with an average of 1.3 passes (0–4). More than two passes were performed in six targets (6.7 %). pREset LITE 4–20 was attempted in 57, pREset LITE 3–20 in 25, and both devices in 8 targets. On two occasions microcatheter passage of the occlusion was possible but vessel tortuosity precluded placement of pREset LITE 4–20 due to high friction. In the first patient intra-arterial thrombolysis was performed without recanalization of the target vessel; in the second case thrombectomy with pREset LITE 3–20 was successful. In 15 of 27 branches with unsuccessful recanalization after thrombectomy with pREset LITE, further treatment was attempted. Seven branches were successfully recanalized by permanent implantation of a stent. Two of the stents were implanted assuming an underlying dissection possibly induced by pREset LITE. Stent retrievers with higher radial force were used in seven targets. In three patients pREset 4–20 was applied leading to recanalization in two. One patient was successfully treated with pREset 6–30. Recanalization with Solitaire 4–20 was attempted in two targets of which one was successfully recanalized. In the last patient recanalization attempts with pREset 4–20, Solitaire 5–20, and Separator 3D remained unsuccessful. In one patient intra-arterial Alteplast was infused but the vessel remained occluded.

**Table 2:** Target branches for thrombectomy with pREset LITE, average target vessel diameter, recanalization results, and number of thrombectomy passes performed with pREset LITE

Target branches for pREset LITE (*n* )	90
M1 segment of the MCA	1 (1.1 %)
Temporal MCA branch	1 (1.1 %)
M2 segment of the MCA	61 (67.8 %)
A2 segment of the anterior cerebral artery	4 (4.4 %)
Pericallosal artery	10 (11.1 %)
Callosomarginal artery	2 (2.2 %)
Frontopolar artery	1 (1.1 %)
Basilar artery	1 (1.1 %)
P1 segment of the posterior cerebral artery	2 (2.2 %)
P2 segment of the posterior cerebral artery	7 (7.8 %)
Average vessel diameter	1.6 mm (1.3–2 mm)
**Recanalization results after thrombectomy with pREset LITE**	
mTICI 0	21 (23.3 %)
mTICI 1	1 (1.1 %)
mTICI 2a	5 (5.6 %)
mTICI 2b	19 (21.1 %)
mTICI 3	44 (48.9 %)
Average number of passes	1.3 (0–4)

*mTICI* modified thrombolysis in cerebral infarction

Possible or definite device-related adverse events consisted of two suspected dissections treated by permanent implantation of a stent. The diagnosis of dissection was anticipated because the vessel remained occluded after thrombectomy and reentry of the occluded vessel was hampered compared with the first catheterization. Both were young patients with a cervical artery dissection as an underlying cause of stroke. One self-limiting extravasation was observed, and in five patients severe vasospasm occurred, which resolved after intra-arterial infusion of vasodilators. In two patients the spread of thrombus material occurred, one was a downstream embolus and one was an embolus into an adjacent branch. We did not observe a clinical deterioration related to these events. Thus, they presumably did not cause additional harm.

Follow-up imaging was available for 74 patients with 87 targets. Per patient it revealed 6 PHII (8.1 %), 5 PHI (6.8 %), and 15 SAH (20.2 %), of which only one was a diffuse SAH. Of these hemorrhages 2 PHI and 12 focal SAH were anatomically related to the pREset LITE target and thus classified as potentially device-related (2.2 % PHI and 13.8 % focal SAH per target). None of these hemorrhages had an impact on the clinical outcome.

**Fig. 3:**
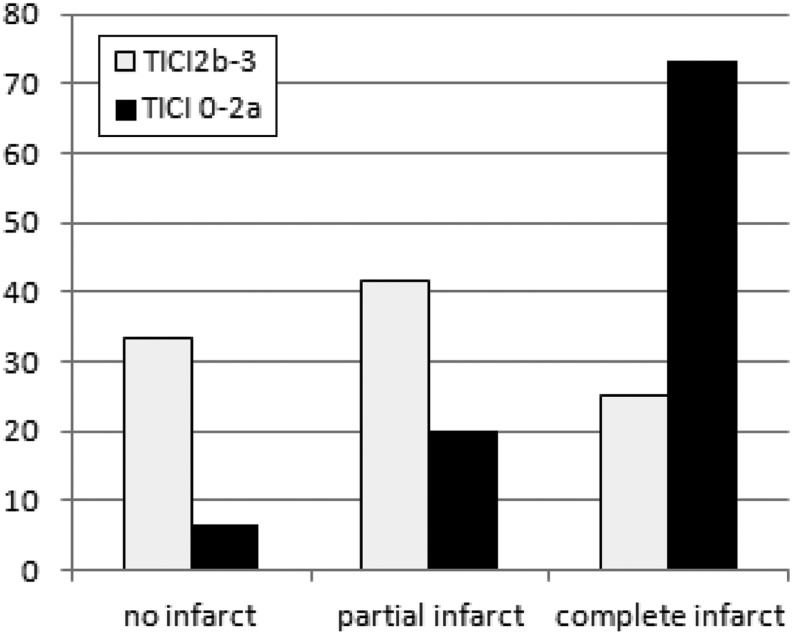
New infarcts in the territory of the target branch depend on recanalization success (*p* = 0.002). With successful recanalization the chance of no infarct is significantly higher compared with a complete infarct (*p* = 0.003, OR 14.7 (95 % CI 1.4–155)) and of a partial infarct compared with a complete infarct (*p* = 0.013, OR 6.1 (95 % CI 1.4–27.6)) but not of no compared with a partial infarct (*p* = 0.627, OR 2.4 (95 % CI 0.2–25.4))

**Fig. 4:**
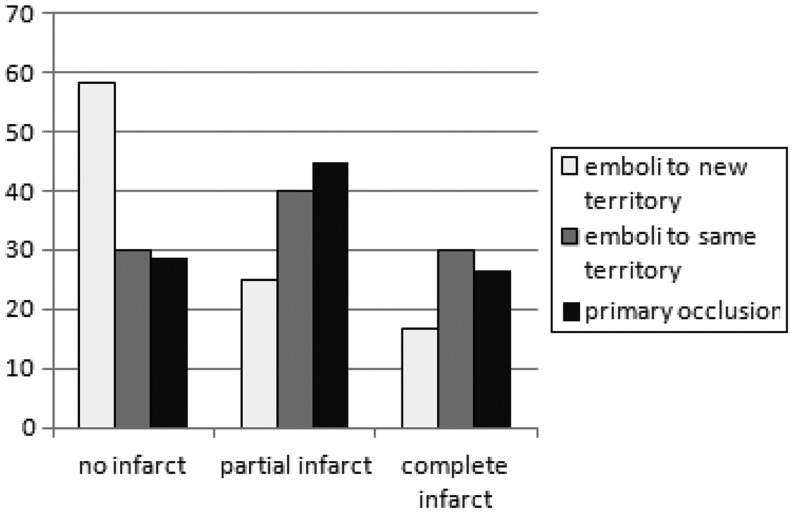
Infarcts on follow-up imaging after successful recanalization of embolic occlusions in a new territory, in the same territory, and primary occlusions. There was a statistically not significant trend for less infarct after successful recanalization of occlusions in a new territory (*p* = 0.438)

Regarding ischemic lesions in the dependent territory, no infarct was seen in 25 (28.7 %), partial infarcts in 33 (37.9 %), and complete infarcts in 29 (33.3 %) occasions. In successfully recanalized patients the rate of no, partial, and complete infarction was 33.3, 45.8, and 25.8 %, respectively. We found a significant relationship between successful recanalization and infarcts on follow-up imaging (*p* = 0.002). Successful recanalization significantly increased the chance of no compared with complete infarction (*p* = 0.003, OR 14.7 (95 %CI 1.4–155)) and partial compared with complete infarction (*p* = 0.013, OR 6.1 (95 % CI 1.4–27.6)) but not of none compared with partial infarction (*p* = 0.627, OR 2.4 (95 % CI 0.2–25.4)) (Fig. 3). Comparing imaging outcome between occlusion types the chance to develop no infarct was highest in patients with emboli into a new territory compared with emboli in the same territory or primary occlusion, but the difference was not statistically significant, neither for the whole cohort nor for successfully recanalized patients only (*p* = 0.163, *p* = 0.438) (Fig. 4). Details regarding recanalization success, type of occlusion, and imaging outcome are summarized in Table 3.

**Table 3:** Extent of infarcts in the small vessel territory depending on the recanalization status for all patients and subdivided into primary occlusions, emboli to the same territory and emboli to a new territory

	*n*	Imaging available *n*	No infarct *n* (%)	Partial infarct *n* (%)	Complete infarct *n* (%)
**All**	90	87			
TICI ≥ 2b pREset LITE	63	62	24 (38.7)	22 (35.5)	16 (25.8)
TICI < 2b pREset LITE	27	25	1 (4.0)	11 (44.0)	13 (52.0)
Otherwise successfully recanalized	10	10	0 (0.0)	8 (80)	2 (20)
All successfully recanalized	73	72	24 (33.3)	30 (41.6)	18 (25.0)
All not successfully recanalized	17	15	1 (6.6)	3 (20.0)	11 (73.3)
**Primary occlusion**	63	61			
TICI ≥ 2b pREset LITE	39	39	14 (35.9)	14 (35.9)	11 (28.2)
TICI < 2b pREset LITE	24	22	0 (0.0)	9 (40.9)	13 (59.1)
Otherwise successfully recanalized	10	10	0 (0.0)	9 (90.0)	1 (10.0)
All successfully recanalized	49	49	14 (28.6)	22 (44.9)	13 (26.5)
All not successfully recanalized	14	12	0 (0.0)	1 (8.3)	11 (91.7)
**Emboli to same territory**	14	13			
TICI ≥ 2b pREset LITE	11	10	3 (30.0)	4 (30.8)	3 (30.0)
TICI < 2b pREset LITE	3	3	1 (33.3)	2 (66.6)	0 (0.0)
Otherwise successfully recanalized	0	0			
All successfully recanalized	11	10	3 (30.0)	4 (30.8)	3 (30.0)
All not successfully recanalized	3	3	1 (33.3)	2 (66.6)	0 (0.0)
**Emboli to new territory**	12	12			
TICI ≥ 2b pREset LITE	12	12	7 (58.3)	3 (25.0)	2 (16.7)
TICI < 2b pREset LITE	0	0			
Otherwise successfully recanalized	0	0			
All successfully recanalized	12	12	7 (58.3)	3 (25.0)	2 (16.7)
All not successfully recanalized	0	0			
**Not classified**	1	1			
TICI ≥ 2b pREset LITE	1	1	0 (0.0)	1 (100.0)	0 (0.0)
TICI < 2b pREset LITE	0	0			
Otherwise successfully recanalized	0	0			
All successfully recanalized	1	1	0 (0.0)	1 (100.0)	0 (0.0)
All not successfully recanalized	0	0			

## Discussion

Triggered by the positive results of the recently published prospective randomized trials, mechanical thrombectomy will become an integral part of stroke therapy and will contribute to improved clinical outcome of patients. However, the trials providing evidence for the effectiveness of endovascular stroke therapy mainly focused on proximal large artery occlusions of either the terminal carotid artery or the M1 segment of the MCA. Despite the fact that some trials allowed M2 occlusions to be included, only a few patients with an occlusion beyond M1 were randomized. This was possibly driven by the assumption that peripheral artery occlusions cause less severe clinical syndromes and respond well to intravenous thrombolysis. Earlier studies proved a relative safety of M2 compared with M1 thrombectomy even with the MERCI device, but still some safety concerns remain [7]. Several curves have to be overcome to access a peripheral target and will be stretched during device retraction. Thus, thrombectomy in small caliber peripheral vessels may harbor an increased procedural risk. The failure to prove a clinical benefit of early M2 reperfusion in a pooled analysis of the PROACT II, IMS, and IMSII study may have supported the reluctance to include these patients into a prospective trial [8].

On the other hand distal vessel occlusions may lead to serious and permanent morbidity. Despite the fact that patients with an M2 occlusion have a better prognosis compared with patients with a more proximal occlusion, unfavorable outcome can be expected in up to 45 % if left untreated [9]. Intravenous thrombolysis improves outcome, but still up to 30 % of patients will die or be dependent at 90 days [10, 11]. Similar considerations hold true for posterior cerebral artery occlusions, which can be associated with severe somatosensory and behavioral as well as cognitive deficits [12]. Even after intravenous thrombolysis the rate of unfavorable outcome after posterior cerebral artery occlusion may be as high as 40 % [13]. These data support the search for more effective treatment strategies for distal vessel occlusions.

Occlusion of distal vessels may also be encountered while treating more proximal targets. Emboli to multiple territories or thrombus extension to small branches can be a primary finding. In addition, thrombus fragmentation and embolization to distal branches or new territories are known side effects of mechanical recanalization. Emboli into new territories can be expected in 0.7–11 % of recanalization procedures depending on the technique applied [14–16]. Recanalization attempts seem reasonable if eloquent brain tissue is jeopardized and recanalization techniques with a good safety profile are available.

Recently some multi- and mono-centric studies emerged addressing thrombectomy of M2 branches as well as anterior cerebral artery occlusions in one series [17–20]. Treatment in these studies was performed with regular stent retrievers and demonstrated good feasibility and safety. Successful recanalization of M2 was associated with improved clinical outcome and smaller infarct size in one investigation [17]. However, in some distal branch occlusions, treatment with already available devices may be considered to be potentially harmful due to the small diameter of the affected vessel, location of the occlusion in a curved vessel segment, or retraction pathway. Retrievers with a reduced radial force and diameter fitting through small caliber microcatheters may increase the spectrum of potentially treatable vessel occlusions.

pREset LITE 4–20 and 3–20 were designed and approved to meet these demands. In this manuscript we report our clinical experience with the two devices regarding recanalization results and safety data. pREset LITE was selected in situations in which the operator felt treatment with regular devices to be associated with an increased risk. In our analysis we included target branches with a diameter equal to or less than 2 mm to focus on vessels, which cannot be treated with the well-described and established regular devices according to their specification.

Accessibility of peripheral branches was excellent, with only 2.2 % failed attempts and successful recanalization was achieved in 70.0 % of targets. For the treatment of proximal vessels with the regular pREset retriever a recanalization rate as high as 85 % was previously reported [21]. The small gap of successful recanalization between proximal and distal vessels could possibly be explained by the reluctance to act aggressively in small branches. This is underlined by the finding that more than two thrombectomy passes were rarely performed in a distal vessel. Recanalization rates reported in the recently published randomized trials addressing thrombectomy of large vessel occlusion ranged between 59 and 88 %, which compares well with the 70 % achieved using pREset LITE in small vessels [1–5].

In terms of adverse events, significant vasospasm after retraction of the device was observed in 5.6 % but always resolved with the infusion of vasodilators and did not affect outcome in any case. Cerebral vasospasm occurs frequently after mechanical thrombectomy. A detailed analysis of procedural complications in the “Solitaire With the Intention For Thrombectomy” trial revealed some vasospasm in 22.5 % after treatment with Solitaire but none was symptomatic [12]. Haussen et al. reported their experience with Trevo XP 3 × 20 for the treatment of distal vessels and described relevant vasospasm in five of eight patients, and vasospasm always responded to vasodilators [22]. Thus, vasospasm after thrombectomy seems to be a benign phenomenon even when treating smaller distal branches.

New emboli after thrombectomy with pREset LITE occurred only in two occasions. Theoretically, the risk of losing thrombus material using small, low radial force retrievers loaded with small thrombi could be increased during the passage of larger proximal vessel segments. Since all patients in this series were treated with intermediate catheters and distal aspiration, we did not observe emboli into new but only the same territory. Emboli into the same vascular territory have been described to occur in approximately 4.4 % of cases [12]. Using this as a benchmark, a frequency of 2.2 % after treatment with pREset LITE is acceptable.

In two targets (2.2 %) dissection was suspected because the vessel remained occluded after thrombectomy and reentry seemed more difficult compared with prior catheterizations. No further thrombectomy was performed to avoid harm and, instead, self-expanding stents were permanently implanted to restore flow. Target vessel dissection is rarely reported after stent-retriever thrombectomy with a maximum rate of 1.5 % [15]. A rate of 2.2 % after treatment of distal vessel branches is slightly higher, but both intracranial dissections occurred in patients with a cervical artery dissection and may reflect a higher vulnerability due to an underlying vessel wall disease. In addition, the diagnosis of dissection was only suspected based on the difficulty to reenter the target branch and was not proven by the typical aspect of an intimal flap. A different explanation of this phenomenon (e.g., change of the mechanical properties of the thrombus due to the previous recanalization attempt) cannot be excluded. Alternatively, dissections may be underreported in the literature and “hiding” in the rate of unsuccessful recanalization attempts.

With the treatment of distal branches the risk of vessel perforation and intraprocedural hemorrhage could be increased. Device-related intraprocedural contrast extravasation was only observed once; it was self-limiting and without impact on the final outcome. Analysis of posttreatment imaging revealed potentially device-related PHI or SAH in 2.2 and 13.3 %. Although none of the latter altered the clinical course, the frequency of SAH was in the upper range compared with literature references. SAH was reported to occur in 0.9–16.2 % in stent-retriever studies and was below 10 % in most publications [1–5, 12, 15, 23]. Studies explicitly analyzing the frequency and impact of SAH or contrast extravasation after thrombectomy performed imaging immediately or a few hours after treatment, and SAH may disappear at later time points [22, 24]. Since our patients did not routinely receive immediate posttreatment imaging studies, the frequency of SAH may even be underestimated. The authors reporting the highest rate of SAH of 16.2 % found an association with rescue angioplasty, which cannot serve as an explanation for the SAH we found after thrombectomy of small branches. Therefore, the hemorrhage is most likely attributable to the distal location of the target vessel. Stretching and rupture of small perforating branches may serve as an explanation for these angiographically occult bleedings. Predictors of procedure-related SAH were analyzed in detail after treatment with the MERCI device, and the authors found distal MCA occlusion to be an independent predictor together with hypertension, rescue angioplasty, and intraprocedural perforation [25]. In the same study SAH was without impact on clinical outcome unless extensive or accompanied by severe parenchymal hematoma. The benign course of SAH due to angiographically occult bleeding was also confirmed in a retrospective case control study investigating this phenomenon after stent-retriever thrombectomy [26]. In summary, up to now there is no evidence of clinical impairment due to focal SAH after mechanical thrombectomy, but some uncertainty remains since larger studies are still lacking.

Statistical analysis revealed a significant relationship between recanalization and infarcts in the dependent territory. With successful recanalization the chance to develop none or only a partial infarct was higher compared with the risk of complete infarction. Haussen et al. presented their initial experience using “Baby Trevo” in distal intracranial vessels [16]. The number of patients treated in their series was limited (*n* = 8), but all targets were successfully recanalized. Only one patient developed a complete infarct, four had a partial infarct, and three no infarct. Thus, the results were qualitatively comparable with the findings in our study. Due to the fact that the majority of patients developed at least a partial infarct, the authors considered the effectiveness of thrombectomy in distal vessels to be unproven. Our study had a higher overall number of patients and a reasonable number of not successfully recanalized targets, which served as a comparator. Under these premises we were able to demonstrate that successful recanalization significantly reduces the risk to develop a complete infarction in the dependent territory, and partial infarction also reflects at least partial treatment success. In addition, we found a trend for less infarcts in patients recanalized for emboli into a new territory. This finding is most likely attributable to the shorter occlusion time compared with patients with primary occlusions or emboli into the already affected territory.

Our study is limited by its retrospective nature and the fact that efficacy was proven by infarct occurrence and size, which served as a surrogate for clinical relevance. Further studies to directly address the impact on clinical outcome are warranted and could potentially be conducted in cases with primary isolated small branch occlusions. With embolic occlusions of more proximal vessels extending into smaller branches or secondary emboli, the impact of recanalization of a single branch on clinical outcome will be difficult to determine.

In summary, recanalization of distal branches is double-edged with a good chance for successful recanalization and reduction of infarct size but an increased risk of focal SAH. Up to now there is no evidence for a relevant clinical impact of focal SAH after thrombectomy, but larger confirmative studies are lacking. With the remaining uncertainty, the most reasonable approach would be to confine thrombectomy of small vessels to branches supplying eloquent brain tissue, which increases the likelihood of a relevant clinical benefit.

## Conclusion

Mechanical thrombectomy in small vessels with pREset LITE is feasible with good recanalization and reasonable safety margins. Successful recanalization of small branches significantly reduces the risk of infarction in the dependent territory. The impact on the overall clinical outcome remains to be determined.
